# Indolent T-Cell Lymphoproliferative Disorders of the Gastrointestinal Tract (iTLPD-GI): A Review

**DOI:** 10.3390/cancers13112790

**Published:** 2021-06-03

**Authors:** Francesca Sanguedolce, Magda Zanelli, Maurizio Zizzo, Stefano Luminari, Giovanni Martino, Alessandra Soriano, Linda Ricci, Cecilia Caprera, Stefano Ascani

**Affiliations:** 1 Pathology Unit, Policlinico Riuniti, University of Foggia, 71121 Foggia, Italy; 2 Pathology Unit, Azienda USL-IRCCS di Reggio Emilia, 42122 Reggio Emilia, Italy; Magda.Zanelli@ausl.re.it; 3 Surgical Oncology Unit, Azienda USL-IRCCS di Reggio Emilia, 42122 Reggio Emilia, Italy; Maurizio.Zizzo@ausl.re.it; 4 Clinical and Experimental Medicine PhD Program, University of Modena and Reggio Emilia, 41121 Modena, Italy; 5 Hematology Unit, Azienda USL-IRCCS di Reggio Emilia, 42122 Reggio Emilia, Italy; Stefano.Luminari@ausl.re.it; 6 Hematology Unit, University of Perugia, CREO Perugia, 06124 Perugia, Italy; gio.martino@gmail.com; 7 Gastroenterology Unit, Azienda USL-IRCCS di Reggio Emilia, 42122 Reggio Emilia, Italy; Alessandra.Soriano@ausl.re.it; 8 Pathology Unit, Azienda Ospedaliera S. Maria di Terni, University of Perugia, 05100 Terni, Italy; lindaricci1987@hotmail.it (L.R.); ceciliacaprera@libero.it (C.C.); s.ascani@aospterni.it (S.A.)

**Keywords:** indolent T-cell lymphoproliferative disorder of the gastro-intestinal tract (iTLPD-GI), diagnosis, immunohistochemistry

## Abstract

**Simple Summary:**

This review aims to better define the clinical, pathological, and molecular features of the novel lymphoproliferative disease termed “indolent T-cell lymphoproliferative disorder of the gastro-intestinal tract (iTLPD-GI)”, to discuss potential pitfalls in differentiating this entity from other neoplastic and non-neoplastic disorders arising at the same site, and to point out a biomarker-based approach to the diagnosis.

**Abstract:**

iTLPD-GI is a low-grade clonal T-cell lymphoproliferative disease arising in GI organs. It is an uncommon disease, and only recently has it been enlisted as a distinct provisional entity in the current WHO Classification. Data from the literature disclose high heterogeneity in terms of pathological and molecular features; on the other hand, establishing an accurate diagnosis of iTLPD-GI is of pivotal importance, since treatment options are different from that of other, more frequent lymphomas that arise in the gastrointestinal tract. In this review, we aimed to better define this novel entity, and to identify useful diagnostic biomarkers; moreover, we provide a biomarker-based approach to the diagnosis and describe the most common issues in differentiating iTLPD-GI from other neoplastic and non-neoplastic disorders.

## 1. Introduction

Indolent T-cell lymphoproliferative disorder of the gastro-intestinal tract (iTLPD-GI) is a low-grade, clonal, non-epitheliotropic T-cell lymphoproliferative disease, consisting of small lymphocytes, which can affect any site in the GI tract, most commonly the small bowel and colon [1,2]. It probably arises from lamina propria lymphocytes [2].

This disorder has been recently included as a provisional entity in the revised fourth WHO classification of lymphoid neoplasms [2], with rare, previous descriptions encompassing small case series and single case reports [3,4,5,6,7,8,9,10,11,12,13,14,15,16,17]; fewer than 80 cases have been reported in the literature to date [3,4,5,6,7,8,10,11,12,13,14,15,16,17,18,19,20]. According to the available literature, iTLPD-GIs feature high heterogeneity in terms of pathological and molecular features [3,12,17,21].

In order to better define this novel entity, and to identify useful diagnostic biomarkers, we performed a systematic review of the literature and presented its results in three sections: first, we detail the clinical and pathological updated features of iTLPD-GI, then we provide a biomarker-based approach to the diagnosis, and finally we describe the most common issues in its differential diagnosis.

## 2. Materials and Methods

This systematic review adhered to the guidelines proposed by the Preferred Reporting Items for Systematic Reviews and Meta-analyses (PRISMA) [22]. A literature search was conducted using PubMed, Scopus, and Google Scholar databases with the following keywords: ‘‘indolent T-cell lymphoproliferative disorder AND gastrointestinal tract’’, “iTLPD-GI”. The search was performed from the inception of the databases until July 1, 2020. The inclusion criteria were as follows: (1) retrospective, observational case-control studies, case reports and/or series, literature reviews; (2) presence of clinical, pathological, immunohistochemical, and/or molecular findings. The exclusion criteria were as follows: (1) studies without full text; (2) studies not published in English; (3) lack of adherence to the diagnostic criteria for iTLPD-GI according to the latest WHO Classification of Haematopoietic and Lymphoid Tumours [2]. After reviewing the titles and the abstracts, two independent reviewers (FS and MZ) ascertained whether they met inclusion criteria by reading exhaustively the full-text articles. A third author (SA) resolved eventual discrepancies. Data extraction from the eligible studies included for the systematic review was performed independently. The following information was collected from each study: first author’s name, journal, publication date, clinical and pathological findings, and the immunohistochemical profile of each reported case. The retrieved data were prepared in a custom Microsoft Excel file for further evaluation and division into groups.

## 3. Updated Clinical and Pathological Findings on iTLPD-GI

### 3.1. Epidemiology and Aetiology

The disease usually presents in adulthood, although the age at diagnosis ranges between 15 and 77 years (median 51 years) [6,12,14,16,17], and occurs slightly more commonly in males than females (M:F—1.5:1) [6,12,14,16,17,23].

The aetiology of iTLPD has not yet been disclosed. Some cases have occurred in patients with a history of inflammatory bowel disease (IBD), such as Crohn’s disease, autoimmune disorders (autoimmune enteropathy, rheumatoid arthritis), and viral infection (HSV, HHV6, HTLV1) [12,14].

### 3.2. Clinical Features and Course

Signs and symptoms at diagnosis are relatively non-specific, namely, diarrhoea, weight loss, abdominal pain, dyspepsia, vomiting, and GI bleeding [6,12,14,16,24]; therefore, many cases have been misdiagnosed as non-responsive or refractory celiac disease (RCD), IBD, or irritable bowel syndrome (IBS) [3,4,5,6,7,8,10,11,12,13,14,15,16,17,18,19,20]. Prior misdiagnoses of seronegative RCD in a high proportion (55%) of patients were due to misinterpretation of the histopathologic changes and defective laboratory testing [21]. In a very few cases, iTLPD-GIs were detected incidentally in asymptomatic patients [6,21].

The small bowel and colon are the usual primary sites, while the stomach, oesophagus, and oral cavity are less commonly involved; the disease extent is variable, from localized to multifocal lesions or infiltrates in one or more organs, either adjacent or not [6,12,14,16,23]. Endoscopic findings show normal to nodular and/or polypoid, hyperaemic mucosa, sometimes displaying superficial erosions and ulcers [6,7,12,14,16]. Peripheral lymphadenopathy is absent; however, some patients may present with mesenteric lymphadenopathy [11,12,13,14,15,16], with mild fluorodeoxyglucose (FDG) uptake at positron emission tomography (PET) scan [12,15,16]. Liver, bone marrow, and peripheral blood involvement has been infrequently described [7,10,11,13,14,16,25].

The vast majority of patients have an indolent and protracted clinical course, lasting for years to decades, with persistent disease and/or occasional relapses (chronic relapsing clinical course) [3,4,5,6,7,8,9,10,11,12,13,14,15,16,17,26]; most iTLPD-GIs have limited or no response to conventional chemotherapy [3,6,7,10,11,12,14,15,16,24,25], and it has been suggested that localized forms may benefit from involved-field radiotherapy (IFRT) [27].

Disease progression and transformation to an aggressive lymphoma (namely, peripheral T-cell lymphoma, not otherwise specified (PTCL, NOS) or anaplastic large cell lymphoma (ALCL)) has occurred in less than 10 patients, generally after several years [7,16,17,21,25].

### 3.3. Pathology

**Figure 1:**
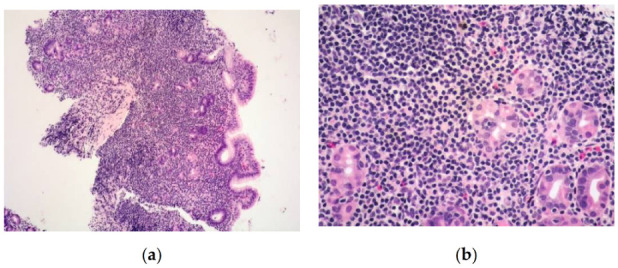
A gastric biopsy featuring diffuse infiltrate of the lamina propria and, focally, the submucosa (**a**); at higher magnification, lymphoid cells are small, monomorphic, with bland cytology (**b**). (Haematoxylin and eosin, original magnification 200× and 400×).

There is a characteristic variably dense, monomorphous, lymphoid infiltrate without destruction, which is usually confined to the lamina propria, where it usually segregates and deforms glands or crypts [1,2,7,12,14,16,28], and occasionally spreads into the muscularis mucosae and submucosa [1,2,7,12,16] in the absence of well-defined masses and full thickness wall involvement [12]. The infiltrate consists of small to occasionally medium-sized lymphocytes exhibiting a mature phenotype, with round or slightly atypical nuclei, normal-appearing chromatin, very small nucleoli, and a low amount of cytoplasm [1,2,7,12,14,16] (Figure 1a,b); the Ki-67 proliferation index is very low, being <10% in all cases and mostly <5% [1,2,12,16]. The lymphoid infiltrate expresses CD3, and may be CD4+CD8− and, less commonly, CD4−CD8+ [1,2,7,12,16,17]. CD4 expression alone has been documented in almost two-thirds of the reported cases so far, while both CD4−CD8− (double negative) and CD4+CD8+ (double positive) cases have been rarely described [12,17,21,23,28,29]; some authors questioned whether these constitute bona fide examples of iTLPD-GI.

There may be some eosinophils [7,12,16], as well as scattered lymphoid follicles and non-necrotising granulomas; actually, the latter have been reported in 5% of all cases [7,14,23]. Angioinvasion/destruction and necrosis are absent [7,14].

A diffuse increase in intraepithelial lymphocytes is uncommon, yet clusters of lymphocytes can be observed within the crypt epithelium (epitheliotropism, or lymphoepithelial lesions) [1,2,7,12,16,23]; however, in some CD4+ cases lymphocyte infiltrates are present in the bottom parts of the villi and crypts [14,16]. The profile of small bowel villi ranges from normal to atrophic; the latter is an infrequent finding [7,14,16]. Conversely, crypt hyperplasia is evident [23].

High-grade transformation has been rarely reported, showing large and pleomorphic infiltrating cells, with morphologic features that may be indistinguishable from PTCL, NOS, enteropathy-associated T-cell lymphoma (EATL), or ALCL [7,16,17,21,25].

### 3.4. Molecular Genetics

All cases of iTLPD-GIs show clonal rearrangement of T-cell receptor (TCR) genes, either TCRβ or TCRγ [12,14,16,17].

Several non-recurrent changes have been described by genome-wide DNA copy number analysis [7,14,16,18], as well as IL2 and/or TNFRSF17(BCMA) gene alterations in single cases [14,16,30]. Two out of four (50%) of the CD8+ cases in the series by Soderquist et al. [21] exhibited structural alterations involving the 3′ untranslated region of the IL2 gene.

No activation of STAT3 signalling [12] was reported in a previous series of five CD8+ iTLPD-GIs assessed by Sanger sequencing for STAT3 SH2 domain hotspot mutations or phosphorylated (p)-Y705-STAT3 expression. However, a recent study demonstrated recurrent STAT3-JAK2 rearrangements by FISH in four out of five patients with CD4+ iTLPD-GIs, with evidence of STAT5 activation on staining for pY694-STAT5 [17]; three of their cases showed STAT3-JAK2 fusion with identical breakpoint. In keeping with these findings, one out of three CD4+ cases reported by Montes-Moreno et al. [24] demonstrated JAK2 breaks and STAT3-JAK2 fusion, and mutations in the JAK-STAT signalling pathway genes were observed in 75% of the CD4+ cases and in the CD4+/CD8+ and CD4−/CD8− cases reported by Soderquist et al. [21]. Such findings would suggest that iTLPD-GI is a heterogeneous group, as regards its molecular profile, with STAT3-JAK2 fusion being reported in CD4+/CD8− cases only; however, the currently available evidence is not strong enough to support this hypothesis; further studies on larger case series are warranted. Deletion of SOCS1, a negative regulator of the JAK family proteins [31], which was seen in a colonic CD4+ iTLPD [21], is a recurrent abnormality in a variety of T-cell lymphomas and more commonly reported in mycosis fungoides [32]. As disorders in JAK-STAT signalling pathway genes are a very common finding in many forms of T-cell lymphoma, mostly with a cytotoxic phenotype, current evidence suggests that they may have a possible role in the pathogenesis of this disorder [17,21,33]; however, such alterations were not seen in the CD8+ cases of the series [21], which share a cytotoxic phenotype with many of those T-cell lymphomas. Interestingly, mutations in the JAK-STAT pathway have been reported in a recent series of NK-cell enteropathy [34]. It has been suggested that the assessment of JAK2 rearrangements by FISH may be a useful adjunct to the diagnosis and differential diagnosis of these lesions [17].

Recurrent and novel genetic abnormalities involving epigenetic modifier genes (TET2, DNMT3A, KMT2D) in different immunophenotypic subtypes of iTLPD-GIs were reported in a recent series of 10 cases [21].

Transformation to high-grade disease may be associated with the gain of complex chromosomal changes [16]. 

### 3.5. Prognosis and Treatment

The disease tends to have a chronic course, with low morbidity and lasting survival, usually exceeding 10 years [7,12,14,16]. There is no agreement over the treatment of iTLPD; so far, steroid administration has been shown to be more effective than conventional chemotherapy [7,12,14,16]. It has been suggested that CD4+ cases may have a different pathogenesis and carry a higher risk of progression, yet data so far are very limited [7,16,17,24].

## 4. Immunophenotypical Profile of iTLPD-GI

**Figure 2:**
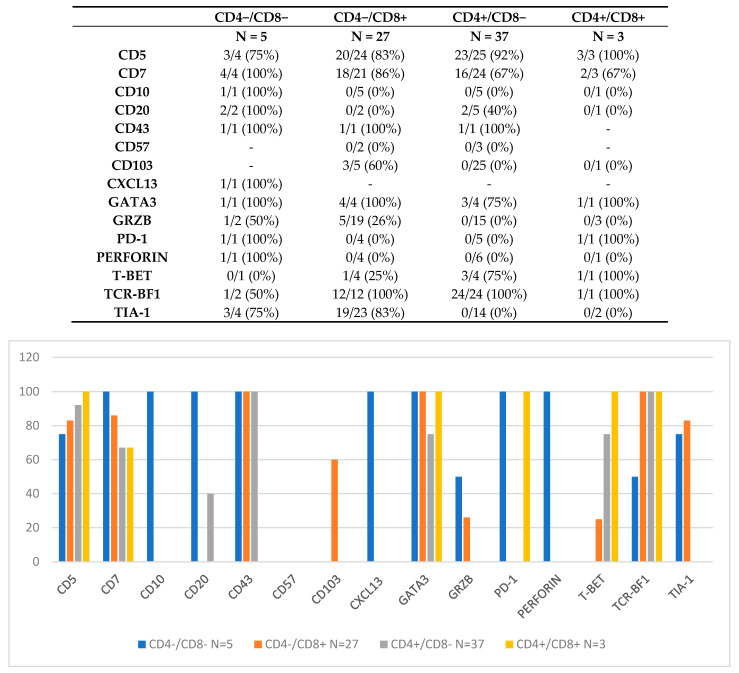
Immunoprofile of the 4 groups of iTLPD-GIs, stratified according to their coordinate CD4/CD8 expression.

The results from our analysis of the literature are described in Figure 2. On the basis of the coordinate expression of CD4 and CD8, four groups have been identified, namely CD4−/CD8−, CD4−/CD8+, CD4+/CD8−, and CD4+/CD8+; the immunohistochemical profile of each group has been detailed, and some differences have emerged among the groups.

### 4.1. Pan-T-Cell Markers

**Figure 3:**
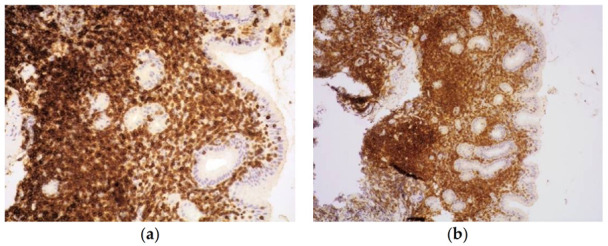
Intense and diffuse expression of T-cell markers CD3 (**a**) and CD4 (**b**). (Haematoxylin and eosin, original magnification 400× and 200×).

The pan-T-cell markers CD2, CD5, and CD7 are usually expressed (Figure 3a,b), yet downregulation or loss of CD5 or CD7 may be seen in some cases [7,8,11,12,13,14,16,17]. 

CD7 is the earliest T-cell marker, in that it is present on early T-lymphocytes and thymocytes; its expression persists in the majority of mature T-lymphocytes. CD5 is a glycoprotein receptor expressed in the majority of T-lymphocytes and a subset of B-lymphocytes. Both may be progressively decreased or lost in T-cell lymphomas. Interestingly, high levels of CD5 expression have been reported in all groups of iTLPD-GI; on the other hand, CD7 positivity is more frequent in CD4− than in CD8− cases, which may provide a useful clue in the diagnosis of these subsets of iTLPD-GIs.

### 4.2. TFH Markers

CD10, PD1, and CXCL13 are markers of T-follicular helper (TFH) cells. Although there is limited data regarding their expression in iTLPD-GI (11, 10, and 1 cases, respectively), most positive cases belong to the CD4−/CD8− group (1 CD10+ CXCL13+ case [24] and 1 PD1+ case [21]). Another PD1+ case was CD4+/CD8+. Due to their morphologic, immunophenotypic, and clinical features, iTLPD-GIs have been postulated to be part of the spectrum of primary cutaneous CD4+ small/medium sized pleomorphic T-cell lymphomas, which are thought to be of TFH cell origin [15,16,33]. Most cases were negative for TFH markers [11,15,16,28]; however, it cannot be ruled out that CD4+ iTLPD-GIs may arise from a particular TFH subtype, or another functional subset or lineage of CD4+ T-cells [16,35].

### 4.3. Cytotoxic Markers

TIA-1 is expressed in most CD4− cases, both CD8− and CD8+ (75% and 83%, respectively), and in three out of four tested cases with subsequent transformation to high-grade lymphoma (the three of them being CD4−/CD8+). Positivity for granzyme B is far lower in both groups, and negativity for both cytotoxic markers has been reported in all CD4+ cases [12,13,17,26,28,36]. These findings highlight the striking similarities between CD8+ iTLPD-GIs and indolent CD8+ lymphoid proliferations of the ear/primary cutaneous acral CD8+ T-cell lymphomas, which affect mostly males [37,38,39]. Matnani et al. suggested that the cell of origin of CD8+ iTLPD-GIs might express a latent cytotoxic phenotype [36].

### 4.4. Clonality Markers

The antibody clone βF1 maps to an epitope within the constant region of the TCR-β, present only within αβ and not γδ T-cells [40]. Most cases (98%) were TCR-βF1+, including all cases belonging to the CD4−/CD8+, CD4+/CD8−, and CD4+/CD8+ groups [1,2,12,14,16,17].

### 4.5. CD103

The integrin protein CD103 is expressed on intraepithelial lymphocytes T-cells and on some peripheral regulatory and lamina propria T-cells [41,42]. Lack of CD103 expression has been reported in most iTLPD-GIs [7,9,11,15,28], with exceptions belonging to the CD4−/CD8+ group (three out of five cases, 60%) [6,14,21,36]; due to its presence in at least a subset of CD8+ cases, Matnani et al. [36] suggested an origin of these iTLPD-GIs from a mucosal CD8+ T-cell precursor. Moreover, it has been hypothesized that CD103+ iTLPD-GIs could arise either from specific integrin expressing lamina propria T-cells [43] or through activation induced upregulation of CD103 [44,45]. Interestingly, one CD103+ CD8+ iTLPD also showed focal CD56 expression [21] and was initially diagnosed as MEITL; it has been reported that this patient is alive with disease after seven years. 

### 4.6. CD30

CD30 was negative in all tested cases [12,14]; however, three cases acquired CD30 expression upon large cell transformation of the neoplastic cells [16,17].

### 4.7. T-Bet and GATA3

The transcription factors T-box expressed in T-cells (T-bet) and GATA-binding protein-3 (GATA3) act by regulating the differentiation of naïve T-helper cells towards Th1 or Th2 cells. Their coordinate expression resulted in Th1, Th2, or hybrid Th1/2 profiles within the CD4+ and CD4+/CD8+ iTLPD-GIs reported by Soderquist et al. [21]. This last hybrid profile of mucosal Th1/2 cells shows striking similarities to those described in primary parasite-induced immune responses [46]; alternatively, it may stem from either Th1 or Th2 cells having undergone cytokine mediated reprogramming to acquire a hybrid phenotype [47]. The phenotypic switch from a Th1/Th2 to Th2 profile, which has been described in one CD4+ case with time [21], points to possible lineage and/or functional adaptability, at least in selected cases. Moreover, GATA3 modulates several functions of CD8+ T-cells, including their activation and cytolytic activity [48]. The role of T-bet/GATA3 co-expression in CD8+ iTLPDs has not been clarified yet. GATA3 expression demonstrated prognostic impact in PTCL, NOS according to previous studies [49,50]; in keeping with these findings, all tested iTLPD-GIs undergoing high-grade transformation (1 CD4+/CD8− and 2 CD4−/CD8+) were GATA3 positive [21].

## 5. Differential Diagnosis

**Figure 4:**
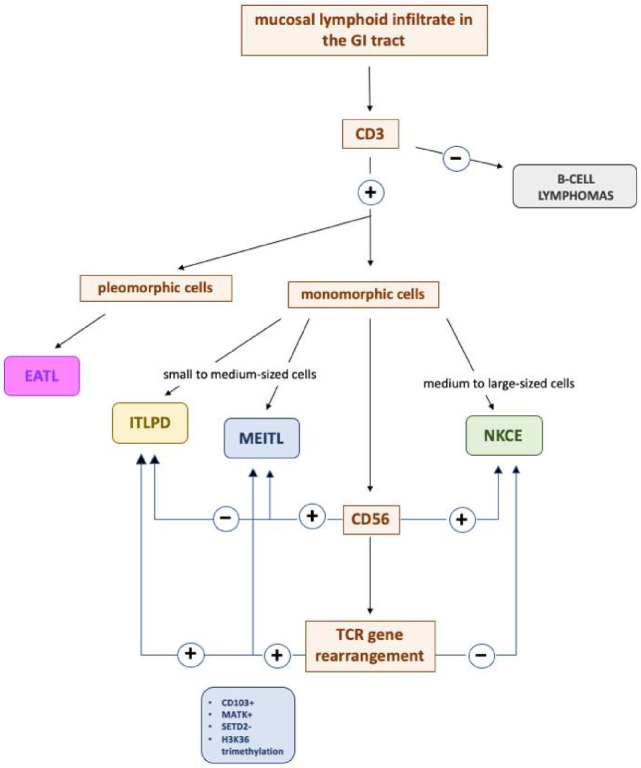
Essential diagnostic algorithm to discriminate iTLPD-GI from its closest mimicries.

The diagnosis of iTLPD-GI can be challenging due to their rarity, the presence of overlapping histopathologic features with both aggressive lymphomas and inflammatory conditions, and the occasional occurrence of non-conventional immunophenotypic features. Therefore, a definite diagnosis should be rendered only when all clinical, endoscopic, and radiological information has been acknowledged, since the presence of mass lesions and involvement of the full wall thickness may not be evident at endoscopic biopsy [51]. Moreover, in limited biopsies, definitive distinction from EATL or other types of T-cell or NK-cell lymphomas (such as ENKTL and PTCL, NOS) may not be possible due to limited material and crush artifacts [23,52]; thus, it has been hypothesized that some iTLPDs were previously misclassified as aggressive intestinal T-cell lymphomas because of the relatively recent recognition of these disorders as distinct entities. In a recent study, as many as 40% of the iTLPDs had been previously misdiagnosed as EATL and/or MEITL [21]. In this section, we list the most common entities in the differential diagnosis of iTLPD-GI; a brief algorithm is provided in Figure 4.

### 5.1. iTLPD-GI vs. MEITL

Distinguishing CD103+ iTLPD-GI cases from MEITL can be challenging, since both entities show a dense lymphoid infiltrate of small to medium-sized lymphocytes (although those in iTLPD-GI tend to be smaller), and information about the clinical presentation and course may be very important in this setting. Patients with MEITL, as well as with EATL, usually present with a mass lesion or destructive growth pattern, and acute symptoms related to intestinal obstruction and perforation [36,53,54,55,56].

A diagnosis of iTLPD-GI is supported by lamina propria-confined neoplastic cells with bland cytology, a low Ki-67 index, and lacking CD56 and MATK expression [21,57]. Assessing SETD2 and H3K36me3 expression may also be of help in the differential diagnosis between ITLPDs and MEITL, since the latter often shows loss of SETD2 and H3K36 trimethylation [58]. The two entities have striking differences in clinical outcome, since patients with MEITL have a median survival of <1 year, and response to initial treatment may portend longer overall and progression-free survival [53]; therefore, a misdiagnosis would lead to unnecessary aggressive treatment [59]. In addition, 8q gain, a known recurrent abnormality in MEITL [60], was observed in one case of iTLPD-GI [16].

### 5.2. iTLPD-GI vs. EATL

Enteropathy-associated T-cell lymphoma (EATL) is a distinct entity of primary intestinal T-cell lymphoma with aggressive progression, displaying pleomorphic intermediate to large-sized cells with round or angulated vesicular nuclei, prominent nucleoli, and moderate to abundant pale-staining cytoplasm in most cases [61,62], with intraepithelial and transmural infiltration of atypical lymphocytes, which are usually CD4− and CD5−, express cytotoxic granule-associated proteins, and have a high mitotic rate and proliferation index. Moreover, large cell EATLs are CD30+ [62]. 

A recent report pointed out that the NK receptor NKp46, as assessed by immunohistochemistry, is a powerful diagnostic biomarker of EATLs and MEITLs, in comparison to iTLPDs, which were negative in all examined cases [59].

### 5.3. iTLPD-GI vs. NKCE

Both iTLPD-GI and NK-cell enteropathy show low-grade clinical and pathological features and are enlisted among EBER indolent LPDs [63]. Those iTLPD-GIs expressing cytotoxic markers may be easily misdiagnosed as NKCE; however, CD56 expression and lack of TCR rearrangement allow for a prompt distinction between them (Figure 4).

### 5.4. iTLPD-GI vs. ENKTL

Lack of EBV by in situ hybridization (EBER) in all tested cases of iTLPD-GI [1,2,12], along with the absence of angiocentricity or angiodestruction, would argue against a diagnosis of extranodal NK/T-cell lymphoma, nasal type, which can involve the GI tract [20,23,57].

### 5.5. iTLPD-GI vs. B-Cell Lymphomas

Low grade B-cell lymphomas such as extranodal marginal zone lymphoma of mucosa-associated lymphoid tissue (MALToma) and mantle cell lymphoma (MCL) should be listed among possible differential diagnoses, since aberrant CD20 expression has been reported in 4 out of 10 reported cases of iTLPD-GI [20,24,61]. It should be taken into account that this finding might be biased by the underreporting of the vast majority of CD20 negativity. Overlapping elements between iTLPD-GI and MALToma and MCL are the gross features of multiple intestinal polyposis, the presence of lymphoepithelial lesions and monocytoid lymphoid cells, and IRTA1 expression, respectively. A nodular, diffuse or mantle zone pattern along with positivity for cyclin D1 and S0X11 is typical of MCL [2], and the expression of T-cell markers and clonal T-cell receptor management further supports a diagnosis of GI-ITLPD [20].

### 5.6. iTLPD-GI vs. IBD

There are morphological similarities between iTLPD-GIs and IBDs, namely the presence of lymphoid follicles and occasional granulomas, resulting in cases initially misdiagnosed as IBD [36]. Careful histopathologic and immunophenotypic evaluation, in addition the presence of a clonal TCR rearrangement, can distinguish iTLPD from IBD [16]. When present at biopsy examination, crypt distortion along with granulomas in the clinical context of multiple lesions throughout the GI tract may be misleading [7,12,36]. However, the relationship between IBD and indolent T-cell LPD of the GIT is still unclear at present [14,18].

### 5.7. iTLPD-GI vs. CD

Celiac disease (CD) may show some mucosal changes that enter the differential diagnosis of iTLPD; in such cases, the presence of remarkable intraepithelial lymphocytosis and of plasma cells within the lamina propria, in the absence of homogeneous small lymphocytes, points toward the diagnosis of CD rather than iTLPD. The appropriate clinical context, namely lack of CD serologies and associated HLA alleles, further argues against a diagnosis of CD [64] or sprue-like enteropathy [65,66].

## 6. Conclusions

iTLPD-GIs may involve any organ of the GI tract, often as multifocal lesions. The diagnosis of this uncommon entity frequently relies on limited material provided by endoscopic biopsies; therefore, care must be taken in considering the full clinical, endoscopic, and radiological framework and to rule out all the possible morphological mimickers. In this review, we provided a comprehensive clinical, histopathological, immunohistochemical, and molecular picture of iTLPD-GI, discussed its diagnostic biomarkers, and eventually suggested an algorithm to prevent the risk of misdiagnosis.

## Data Availability

Individual patient data from the original studies included in the present review is not available and data sharing at this level is not applicable for a systematic review.
